# The Gospel of Hahnemann

**Published:** 1899-02

**Authors:** A. L. Kennedy

**Affiliations:** Boston, Mass.


### THE GOSPEL OF HAHNEMANN. HOW ARE WE PROCLAIMING IT?

By A. L. Kennedy, M. D., Boston, Mass.

By virtue of circumstances, varying more or less in each individual case, homœopathicians to-day possess the knowledge of certain facts, in regard to the treatment of disease, not generally understood by the medical profession. That we are in some degree responsible for this ignorance on the part of our colleagues is the conviction that prompts this effort.

Nearly a century ago Hahnemann gave to the world the good news of the relief of suffering humanity through gentle means, and such relief as can be found in no other way.

In Hahnemann's day a considerable number availed themselves of his benign method of treatment, and since that time multitudes have enjoyed the blessings of its health-restoring power. How many more might have experienced its benefits if all the so-called homœopaths had been true to Hahnemann's teachings and example!

Does it not seem as if the mere fact of the existence of such a blessing would lead the people to demand that it be given them? In every department in life man is looking for that which is best. Given a thing better than that already in vogue and the people want it and will go after the man who has it. Humanity may be foolish but it is not blind. In the history of reforms the people have shown their readiness to test, to investigate, and on evidence, to accept new views; the people have less prejudices to overcome, fewer theories to be refuted. The members of a profession or calling are proverbially slow to recognize any good that does not come in the legitimate channel—that is, from sources to them oracular. The human mind is naturally skeptical, and, incongruous as it may appear, often becomes more so after years of training and study that are supposed to open up and develop and broaden its powers. To a degree this skepticism may be accounted for from the fact that the individual, owing to the necessity of the case—the brevity of life—cannot personally investigate every question that arises, but must depend upon knowledge gained second-hand from those whose particular experience has led them to become conversant with the facts. And facts even are made to appear in various lights according to the reflecting power of the transmitter. Again there exists also the well-known tendency of man's thought to follow a certain line, to run in a groove, and anything that threatens to disturb such a course is regarded with suspicion. These and many other things might be made excuses for not accepting a truth.

In the medical profession we find no exception. We must take people as we find them; we have no alternative. It has often seemed to us that, if possible, doctors of medicine were more averse to accepting anything not in accord with their pre-conceived ideas than members of any other profession, perhaps, because by the nature of the professional relations they are able, to an exceptional degree, to control the movements, if not the opinions of their patients.

As evidence of this one has but to glance at the history of some of the medical boards during the past few years.

Verily it would seem as if they had constituted themselves dictators to their respective communities, whose members had no rights which they were bound to respect. Indeed, they would fain possess the people, body and soul, and only upon obtaining vouchers duly attested, would the individual be permitted the act of respiration in God's free air! I have nothing against the institution, *per se;* indeed, I regard it as quite necessary. But I must believe the intelligent member of society has some rights that even the average metropolitan board of health is bound to respect. Perhaps no profession exercises such control over its patrons as the medical. This is due in a large part, no doubt, to the universal and widespread ignorance, on the part of the masses, of the laws that govern their physical being.

If along with the study in our public schools of the physiological effects of alcohol upon the human organism, an equal amount of time was devoted to the consideration of the pathological effects of some of the standard drugs in use by the dominant school of medicine, I think it would be of inestimable advantage in warning against their use on the part of the people and a potent factor in limiting their employment by the physicians. There is an altogether too indiscriminate use of drugs that have no legitimate place in any medical armamentarium. Then there is the abuse of drugs which, properly administered, are valuable and effective of good. From the earliest history of medicine to the present day the people have been deluged with drugs sanctioned and advocated by the medical profession. Multitudes of poor unfortunates are thronging our thoroughfares to-day the unconscious victims of this unbridled propensity for drugging. Is it any wonder that daily there come to our offices men and women, and even little children, who bear the marks of this accursed practice! "But the people demand it," one says, "and we cannot stop it." And why do the people demand it? Because they know of nothing better; they are deceived, deluded, ignorant of the injury received by the course they are pursuing. From earliest infancy they are taught by example and precept, not only by parents and guardians but also by the family physician (whom they learn to regard as the personification of wisdom) that this is the thing to do, and that any withholding on their part or deviation from the regular method will be fraught with direful consequences. Thus they go on until, through some accident or chance influence, or, as frequently occurs, through some bitter experience, one and another are aroused to a realization of the fact that there exist some things of which even their family doctor is not aware. We have as much as said that the doctor is the responsible party, in a great measure, for this hindrance to the progress of truth.

If any one questions it he has but to refer to the records of the past; from the earliest history of the art of healing; from the time when the would-be healer performed over his victim his magic and mysterious incantations, until now, any one who dared advocate any new idea or attempt the application of a different principle from that which was sanctioned by antiquity was most summarily dealt with. Surely in the early days it required a brave man to teach a new truth. Such an attempt was deemed sufficient cause for his execution, and this prevailed not alone in the medical profession but was manifest throughout the scientific world in general.

We all know the experience of Hahnemann; how he was driven from his home and suffered persecution in various ways because he advocated that which was destined to become a blessing to mankind. But we need not despair for the world does move, as evidence of which there came first the modification of the punishment meted out to reformers. Instead of being called upon to pay the penalty with their lives these would-be benefactors were subjected to persecutions, varying in kind and degree down through the years, until now a man may advocate a new truth, or present an old truth in a new form without fear of suffering more than the consequences of ridicule or misrepresentation.

We hopefuly anticipate the day when every movement looking toward the improvement of man's condition in any one department of his threefold nature, physical, mental, or moral, will be at least regarded with courtesy till a fair and impartial judgment, born of honest, careful, and wise consideration, shall consign it to the realm of the abandoned or welcome it and gratefully appropriate it as an added proof of the goodness of the Creator.

Now and then there come to us words of refreshing, even as gentle showers upon a thirsty soil, assuring us that our prophecy is not wholly Utopian. Illustration of this is afforded us in the recent utterance of Prof. William James, of Harvard. The quotation is from his address last March, before the Committee of Public Health of the Massachusetts Legislature, in opposition to the restrictive medical bill:

"I rise to protest against this bill; I come to represent no body or persons with special interests, but simply as a private citizen interested in good laws and in the growth of medical knowledge. The medical profession are urging the bill in the interests, as they believe, of true science. Those who oppose it, they think, can do so only in the interests of ignorance and quackery. I hold a medical degree from Harvard University. I belonged, for many years, to the most scientific of our medical societies; I have taught anatomy and physiology, and now teach mental pathology, in Harvard College. The presumption is that I am also interested in science. I am indeed; and it is, in fact, because I see in this bill (along with some good intentions) a movement in favor of ignorance that I am here to oppose it. . . . One would suppose that any set of sane persons interested in the growth of medical truth would rejoice if other persons were found willing to push out their experiences in the mental-healing direction, and provide a mass of material out of which the conditions and limits of such therapeutic methods may at least become clear. One would suppose that our orthodox medical brethren might so rejoice; but instead of rejoicing they adopt the fiercely partisan attitude of a powerful trades union demanding legislation against the competition of the 'scabs.' . . .

"Now as to calling the Massachusetts Medical Society a trades union, trying to influence legislation against scabs, I can hardly imagine any member of the Society affirming that, in the movement of the present bill, trades-union motives are totally absent. I venture to say that you dare not, gentlemen. You dare not convert the laws of this Commonwealth into obstacles to the acquisition of truth. You dare not do it, gentlemen—and yet that is what you are asked to do, exactly, if you pass this bill."

Other equally noteworthy and commendable examples of honesty are not wanting.

Now, gentlemen, in view of the present attitude of society toward medicine and the medical profession, are we using our influence to the best advantage and in every possible way to correct the evils that exist, and to banish from our midst that belief in error that to such a degree permeates the minds of a misguided people? Undoubtedly the best way to drive out the darkness is to let in the light; but should we not in all honorable ways and at every opportunity exert ourselves for the good of the community? With all our good work along professional lines unquestionably the best to-day in therapeutics, it seems to me there should be a more rapid advance of a knowledge of the principle that underlies our practice. To be sure, a good deal is accomplished toward this end by our individual personal instruction to our patients, as we meet them from time to time, and something is done also along the same line by some of our homœopathic colleges (we wish them Godspeed); but these and other means at present in use are not doing the work that ought to be done. All the facilities at present at our command are employed primarily to further other objects, consequently of necessity are inadequate to this particular end. Sometimes I have thought that a course of popular lectures, or a series of popular articles, appearing from time to time in the daily press, might do much to aid in this direction. A few simple statements of fact printed upon slips or leaflets, for discreet distribution, might be of value. All of these and many other like measures have been in use more or less, and undoubtedly have been productive of good, but there remains yet a wide field for occupancy.

Nineteen hundred years ago the Great Physician gave to the children of men His injunction: "Go ye into all the world and preach the gospel to every creature." I believe the spirit of this command, rightly interpreted, conveys the idea that man is to be so taught, respecting the Divine plan concerning the race that no one need be completely under the dominion of evil, but that all may avoid such experience; and this, I believe, old theology to the contrary notwithstanding. The same principle applies in medicine. True, Hahnemann, one hundred years ago, said, "The physician's highest and only calling is to restore health to the sick, which is called healing." But Hahnemann lived in the days when "old school medicine" and "old school theology" held undisputed sway. To-day we recognize the fact that man need not of necessity go to the devil for a number of years, varying according to the prevailing individual tendencies, in order that subsequently he may become converted and be restored to moral health; nor need he become a physical wreck or even extremely ill before the services of the physician may avail.

The time is approaching when, under the benign and far-reaching influence of the gospel of physical healing the homœopathician will find his highest but not his only "calling" consists in the work of instructing those in health in the art of so conserving the forces of their being as to preserve their powers intact. Upon you and me and those whom we represent devolves mainly the duty, as it also becomes the privilege, to hasten the coming of that day.

Upon us, I say, because we represent those who have recognized that in the law of similars is found the science of therapeutics. This beneficent law, given us by the first Great Healer, and promulgated by His devoted and grateful followers from Hahnemann until now, is in itself the guarantee of the approval of the Almighty and therefore of the justice of the cause and of our final victory.

DISCUSSION.

Dr. Close—This is a timely and excellent presentation of genial and cogent philosophy. Just a word as to the promulgation of the truth that is in Homœopathy. It is certainly one of the highest, if not the highest privilege of the homœopathic physician to be a teacher, not only of the evil effects of drugs, and of the abuse of the organism in various ways, but of the principles of Homœopathy, and that is a very broad field. It does not mean the application of the principle of Similia in the selection of a medicine alone, but also in the direction of the life and thought. The principle of Similia, when legitimately carried out, touches every phase and sphere of human life, and furnishes the solution of every problem of reform or reorganization. The homœopathic physician should so fill himself with the spirit of Homœopathy as to become a living evangel of the higher truth.

Dr. Pease—I too, want to thank Dr. Kennedy for his excellent paper, because it is a fine application of the principles I hinted at in my paper yesterday—that is, in regard to the rules of mental and moral hygiene and sanitation that I believe homœopathicians ought to teach. There is no question that people can be helped to understand the evils and ravages of this universal habit of drugging, and prevented from rushing to the thousand and one fads and advertised patent medicines.

Dr. Patch—I would like to applaud Dr. Kennedy's paper most heartily. We ought not to feel superior to our brother physicians, because, I think, physicians are usually earnest and honest in their work. That if they fail the failure may not arise from personal fault. It may be for lack of proper opportunity. I think there is a great deal to be done in the spread of Homœopathy. Every homœopathic physician should be a promulgator of his belief. *The Organon* should be his Bible of practice just as the Bible of Christianity is our great book of faith. There is one grand way in which we may always preach the true gospel. It is by means of the cured case. That is the surest way. Let the people have ocular demonstration in the cured case, and they are immediately convinced that there is something of value behind it.

Dr. Davis—I was exceedingly interested in the paper, and in the remarks that have been made. I think, however, that when the people see the cures we make, they may not be able, unless more light is given them, to distinguish between the true cures we make and the palliation they get from different forms of treatment. They say, "Yes; I have been relieved, but not as quickly as I was when I took Morphine." There seems to be something necessary to give them insight into what accomplishes the cure. That is the great obstacle I frequently meet with. If we have a case of gall-stone or appendicitis we must do something right off or we have failed in their opinion.

Dr. Pease—And yet, Mr. Chairman, in these very cases of appendicitis there comes the opportunity of teaching the injuries of such drugs as Morphine, not only to the patients directly but in obscuring the symptoms which enable us to select and demonstrate the power of the homœopathic remedy. I find that many times I can illustrate a point and carry conviction in just such cases by demonstrating to them that pain is the kindly teacher of nature to show them that there is something wrong.

---

Tight Lacing.—In a paper read before the Georgia Medical Association by Dr. W. E. Fitch, April 21st, the relation of tight lacing to uterine development and disease of the female organs of generation was considered. The author stated that the Africans, Indians, and all other people who wear loose clothing, are almost entirely free from pelvic disorders. They have most natural and perfect pregnancy, the easiest deliveries, and the most satisfactory puerperium.—*Bacteriological Review.*

